# Supplementary material for: Tobacco smoking and smokeless tobacco use among people living with HIV in Zambia: Findings from a 2023 National NCD/HIV Survey
Source: PLoS One. 2025 Jul 14;20(7):e0327130. doi: 10.1371/journal.pone.0327130 (PMC12258560; doi:10.1371/journal.pone.0327130)
Supplement: Attachment — Submitted filename: PONE-D-25-05511_reviewer.pdf [file pone.0327130.s001.pdf]

PLOS ONE

Tobacco smoking and Smokeless tobacco use among People Living with HIV in Zambia Findings from a 2023 National NCD/HIV Survey

--Manuscript Draft--

**Manuscript Number:**	PONE-D-25-05511
**Article Type:**	Research Article
**Full Title:**	Tobacco smoking and Smokeless tobacco use among People Living with HIV in Zambia Findings from a 2023 National NCD/HIV Survey
**Short Title:**	Tobacco smoking and Smokeless tobacco use among People Living with HIV in Zambia
**Corresponding Author:**	Cosmas Zyambo, MD, MPhil, PhD<br>University of Zambia<br>Lusaka, ZAMBIA
**Keywords:**	Tobacco Smoking; Smokeless tobacco; People living with HIV; non-communicable diseases; Zambia
**Abstract:**	Background<br><br>There are significant public health concerns among PLWH who use tobacco as compared to non-users including loss of an average 12.3 life-years. Tobacco users face a higher risk of NCDs such as cardiovascular diseases, hypertension, diabetes mellitus, and non-AIDS-related cancers.<br><br>Aim<br><br>This study investigated factors associated with tobacco smoking and smokeless tobacco use among PLWH in Zambia.<br><br>Methods<br><br>This study used data from a national cross-sectional survey involving 5,204 PLWH from193 clinics across the 10 provinces of Zambia. Tobacco smoking and smokeless tobacco use (SLT), behavior and clinical characteristics were assessed. Logistic regression was used to obtain unadjusted (UOR) and adjusted odds ratios (AOR) at 95% confidence interval (CI).<br><br>Results<br><br>Of the 5,204 PLWH who participated, 9.7% were current tobacco smokers (21.9% men and 3.7% women), 1.4% were smokeless tobacco users (1.81% men and 1.26% women). In multivariable analysis, predictors to tobacco smoking were being males [(AOR of 4.81 (95% CI: 3.36-6.90)]; higher educational attainment levels [(AOR of 0.29 (95% CI: 0.16-0.52)]; alcohol consumption [(AOR of 4.97 (2.93-8.44)]; being overweight or obese [(AORs of 0.55 (95% CI: 0.35-0.85) and (AORs of 0.36 (95% CI: 0.17-0.79)] respectively; non-adherence to antiretroviral therapy (ART) [(AOR of 1.75 (1.14-2.67)]. Predictors to current SLT were average yearly income of >4,000 ZMW [AOR of 0.31 (95% CI: 0.14-0.73)]; alcohol consumption [AOR 14.74 (95% CI: 1.99-109.02)]; non-adherence to ART [AOR of 3.32 (95% CI: 1.54-7.17)].<br><br>Conclusions<br><br>Our findings underscore the need for targeted interventions to reduce tobacco use among PLWH. These include gender-specific programs addressing unique factors for males and females, economic empowerment for low-income females to reduce SLT use, support for ART adherence to lower smoking rates, and alcohol reduction initiatives through counselling and support groups.
**Order of Authors:**	Cosmas Zyambo, MD, MPhil, PhD
	Paul Somwe

Powered by Editorial Manager® and ProduXion Manager® from Aries Systems Corporation

	Chomba Mandyata
	Mwiche Musukuma
	Phoebe Bwembya
	Henry Phiri
	Malizgani Paul Chavula
	Hikabasa Halwindi
	Joseph Zulu
	Wilbroad Mutale
**Additional Information:**	
**Question**	**Response**
**Financial Disclosure**<br><br>Enter a financial disclosure statement that describes the sources of funding for the work included in this submission. Review the submission guidelines for detailed requirements. View published research articles from *PLOS ONE* for specific examples.<br><br>This statement is required for submission and **will appear in the published article** if the submission is accepted. Please make sure it is accurate.<br><br>**Funded studies**<br>Enter a statement with the following details:<br>• Initials of the authors who received each award<br>• Grant numbers awarded to each author<br>• The full name of each funder<br>• URL of each funder website<br>• Did the sponsors or funders play any role in the study design, data collection and analysis, decision to publish, or preparation of the manuscript?<br><br>Did you receive funding for this work?	Yes
Please add funding details.<br>as follow-up to "**Financial Disclosure**<br><br>Enter a financial disclosure statement that describes the sources of funding for the work included in this submission. Review the submission guidelines for detailed	Global Fund

requirements. View published research articles from *PLOS ONE* for specific examples.<br><br>This statement is required for submission and **will appear in the published article** if the submission is accepted. Please make sure it is accurate.<br><br>**Funded studies**<br>Enter a statement with the following details:<br>• Initials of the authors who received each award<br>• Grant numbers awarded to each author<br>• The full name of each funder<br>• URL of each funder website<br>• Did the sponsors or funders play any role in the study design, data collection and analysis, decision to publish, or preparation of the manuscript?<br><br>Did you receive funding for this work?"	
Please select the country of your main research funder (please select carefully as in some cases this is used in fee calculation).<br>as follow-up to "**Financial Disclosure**<br><br>Enter a financial disclosure statement that describes the sources of funding for the work included in this submission. Review the submission guidelines for detailed requirements. View published research articles from *PLOS ONE* for specific examples.<br><br>This statement is required for submission and **will appear in the published article** if the submission is accepted. Please make sure it is accurate.	ZAMBIA - ZM

**Funded studies**<br>Enter a statement with the following details:<br>• Initials of the authors who received each award<br>• Grant numbers awarded to each author<br>• The full name of each funder<br>• URL of each funder website<br>• Did the sponsors or funders play any role in the study design, data collection and analysis, decision to publish, or preparation of the manuscript?<br><br>Did you receive funding for this work?"	
**Competing Interests**<br><br>Use the instructions below to enter a competing interest statement for this submission. On behalf of all authors, disclose any competing interests that could be perceived to bias this work—acknowledging all financial support and any other relevant financial or non-financial competing interests.<br><br>This statement is required for submission and **will appear in the published article** if the submission is accepted. Please make sure it is accurate and that any funding sources listed in your Funding Information later in the submission form are also declared in your Financial Disclosure statement.<br><br>View published research articles from *PLOS ONE* for specific examples.	The authors have declared that no competing interests exist.

Powered by Editorial Manager® and ProduXion Manager® from Aries Systems Corporation

**NO authors have competing interests**<br><br>Enter: *The authors have declared that no competing interests exist.*<br><br>**Authors with competing interests**<br><br>Enter competing interest details beginning with this statement:<br><br>*I have read the journal's policy and the authors of this manuscript have the following competing interests: [insert competing interests here]*<br><br>* typeset	
**Ethics Statement**<br><br>Enter an ethics statement for this submission. This statement is required if the study involved:<br><br>• Human participants<br>• Human specimens or tissue<br>• Vertebrate animals or cephalopods<br>• Vertebrate embryos or tissues<br>• Field research<br><br>Write "N/A" if the submission does not require an ethics statement.<br><br>General guidance is provided below. Consult the submission guidelines for detailed instructions. **Make sure that all information entered here is included in the Methods section of the manuscript.**	Ethics approval was obtained from UNZABREC (REF. 3007-2022). The protocol was subsequently submitted to the Zambia National Health Research Authority (Ref No: NHRA000011/12/09/2022) for registration and approval. Permission was sought from the Ministry of Health to allow data to be collected from the selected health facilities and for their staff to participate in the study.<br>Informed consent was secured from all participants before their involvement in the study. We ensured the confidentiality of the data obtained. Each participant provided written informed consent, facilitated by a trained member of the study team. Potential respondents had the opportunity to ask any questions about the research before deciding whether to participate. Those who agreed to participate were interviewed. Participants received written informed consent forms and information sheets detailing the study, its risks and benefits, and emphasizing the protection of confidentiality

Powered by Editorial Manager® and ProduXion Manager® from Aries Systems Corporation

**Format for specific study types**<br><br>**Human Subject Research (involving human participants and/or tissue)**<br>• Give the name of the institutional review board or ethics committee that approved the study<br>• Include the approval number and/or a statement indicating approval of this research<br>• Indicate the form of consent obtained (written/oral) or the reason that consent was not obtained (e.g. the data were analyzed anonymously)<br><br>**Animal Research (involving vertebrate animals, embryos or tissues)**<br>• Provide the name of the Institutional Animal Care and Use Committee (IACUC) or other relevant ethics board that reviewed the study protocol, and indicate whether they approved this research or granted a formal waiver of ethical approval<br>• Include an approval number if one was obtained<br>• If the study involved *non-human primates*, add *additional details* about animal welfare and steps taken to ameliorate suffering<br>• If anesthesia, euthanasia, or any kind of animal sacrifice is part of the study, include briefly which substances and/or methods were applied<br><br>**Field Research**<br><br>Include the following details if this study involves the collection of plant, animal, or other materials from a natural setting:<br>• Field permit number<br>• Name of the institution or relevant body that granted permission	
**Data Availability**<br><br>Authors are required to make all data underlying the findings described fully available, without restriction, and from the time of publication. PLOS allows rare exceptions to address legal and ethical concerns. See the PLOS Data Policy and FAQ for detailed information.	No - some restrictions will apply

A Data Availability Statement describing where the data can be found is required at submission. Your answers to this question constitute the Data Availability Statement and **will be published in the article**, if accepted.<br><br>**Important:** Stating 'data available on request from the author' is not sufficient. If your data are only available upon request, select 'No' for the first question and explain your exceptional situation in the text box.<br><br>Do the authors confirm that all data underlying the findings described in their manuscript are fully available without restriction?	
**Describe where the data may be found in full sentences. If you are copying our sample text, replace any instances of XXX with the appropriate details.**<br><br>• If the data are **held or will be held in a public repository**, include URLs, accession numbers or DOIs. If this information will only be available after acceptance, indicate this by ticking the box below. For example: *All XXX files are available from the XXX database (accession number(s) XXX, XXX.*).<br>• If the data are all contained **within the manuscript and/or Supporting Information files**, enter the following: *All relevant data are within the manuscript and its Supporting Information files.*<br>• If neither of these applies but you are able to provide **details of access elsewhere**, with or without limitations, please do so. For example:<br><br>*Data cannot be shared publicly because of [XXX]. Data are available from the XXX Institutional Data Access / Ethics Committee (contact via XXX) for researchers who meet the criteria for access to confidential data.*<br><br>*The data underlying the results presented in the study are available from (include the name of the third party*	The data is available if requested from the university of Zambia and the Ministry of Health in Zambia

Powered by Editorial Manager® and ProduXion Manager® from Aries Systems Corporation

and contact information or URL).<br>• This text is appropriate if the data are owned by a third party and authors do not have permission to share the data.<br><br>* typeset	
Additional data availability information:	

Powered by Editorial Manager® and ProduXion Manager® from Aries Systems Corporation

Manuscript

Click here to access/download;Manuscript;Tobacco smoking and Smokeless tobacco use among PLWH -Plos.docx

Tobacco smoking and Smokeless tobacco use among People Living with HIV in Zambia: Findings from a 2023 National NCD/HIV Survey

Authors: Cosmas Zyambo[1], Paul Somwe[2,] Chomba Mandyata[1], Mwiche Musukuma[3,] Phoebe Bwembya[1], Henry Phiri[4], Malizgani Paul Chavula[1], Hikabasa Halwindi[1], Joseph Zulu[5,] Wilbroad Mutale[5]

Affiliations

1. Department of Community and Family Medicine, School of Public Health, University of Zambia.
2. Centre for Infectious Disease Research in Zambia
3. Department of Epidemiology and Biostatistics, School of Public Health, University of Zambia.
4. Ministry of Health, Lusaka, Zambia
5. Department of Health Policy, School of Public Health, University of Zambia.

Corresponding author: Cosmas Zyambo, University of Zambia, School of Public Health; Phone: (+260) 970530106; email: czyambo256@gmail.com

Abstract

Background: There are significant public health concerns among PLWH who use tobacco as compared to non-users including loss of an average 12.3 life-years. Tobacco users face a higher risk of NCDs such as cardiovascular diseases, hypertension, diabetes mellitus, and non-AIDS-related cancers. **Aim:** This study investigated factors associated with tobacco smoking and smokeless tobacco use among PLWH in Zambia. **Methods:** This study used data from a national cross-sectional survey involving 5,204 PLWH from193 clinics across the 10 provinces of Zambia. Tobacco smoking and smokeless tobacco use (SLT), behavior and clinical characteristics were assessed. Logistic regression was used to obtain unadjusted (UOR) and

adjusted odds ratios (AOR) at 95% confidence interval (CI). **Results:** Of the 5,204 PLWH who participated, 9.7% were current tobacco smokers (21.9% men and 3.7% women), 1.4% were smokeless tobacco users (1.81% men and 1.26% women). In multivariable analysis, predictors to tobacco smoking were being males [(AOR of 4.81 (95% CI: 3.36-6.90)]; higher educational attainment levels [(AOR of 0.29 (95% CI: 0.16-0.52)]; alcohol consumption [(AOR of 4.97 (2.93-8.44)]; being overweight or obese [(AORs of 0.55 (95% CI: 0.35-0.85) and (AORs of 0.36 (95% CI: 0.17-0.79)] respectively; non-adherence to antiretroviral therapy (ART) [(AOR of 1.75 (1.14-2.67)]. Predictors to current SLT were average yearly income of >4,000 ZMW [AOR of 0.31 (95% CI: 0.14-0.73)]; alcohol consumption [AOR 14.74 (95% CI: 1.99-109.02)]; non-adherence to ART [AOR of 3.32 (95% CI: 1.54-7.17)]. **Conclusions:** Our findings underscore the need for targeted interventions to reduce tobacco use among PLWH. These include gender-specific programs addressing unique factors for males and females, economic empowerment for low-income females to reduce SLT use, support for ART adherence to lower smoking rates, and alcohol reduction initiatives through counselling and support groups.

Keywords:

Tobacco Smoking, Smokeless tobacco, People living with HIV, Non-communicable diseases, Zambia

Background

The introduction and increased accessibility of combination antiviral therapy (ART) have significantly reduced AIDS-related morbidity and mortality among people living with HIV (PLWH)[1]. However, HIV still claims nearly 1 million lives annually, with the majority of these deaths occurring in sub-Saharan Africa (SSA)[2,3]. While the estimated general population smoke, the prevalence of smoking in PLWH is 2-3 times higher than the general population[4-7], thus posing a significant public health concerns which includes loss of an average 12.3 life-years more as compared to PLWH non-

smokers[7]. PLWH who smoke face a higher risk of non-communicable diseases (NCDs) such as cardiovascular diseases, hypertension, diabetes mellitus, and non-AIDS-related cancers[8]. They are also more likely to be non-adherent to ART, have unsuppressed HIV viral loads, and lower CD4 counts compared to non-smoking PLWH[9-11]. Crothers et al. conducted a study on US veterans, finding mortality rates per 100 person-years to be 1.76 for HIV-negative non-smokers, 2.45 for HIV-positive non-smokers, and 5.48 for HIV-positive current smokers[12]..

Zambia, a lower-middle-income country (LMIC) has one of the highest numbers of PLWH in the sub-Saharan Africa (SSA) region with more than 11% of the adults between 15-49 living with HIV[13]. The prevalence of tobacco smoking has been varying depending on data survey being used. According to the Zambia Demographic Health Surveys (DHS), the overall prevalence of tobacco smokers ranges from 9.9% to 12.0%[14], and 1% among women and 19.6% among men [15]. Global Youth Tobacco Survey (GYTS), which monitors tobacco smoking among school-going adolescents, reports an overall prevalence ranging from 6.8% to 10.5%[16] and the STEPS survey by the WHO in Zambia reveals a tobacco use prevalence range of 8.7% to 12.3%[17]. In all these studies, the rural vs urban and male vs female differentials were significantly observed.

Consistent with findings from other regions, HIV has been associated with increased likelihood of tobacco use in sub-Saharan Africa (SSA), even after adjusting for demographic, socioeconomic, and sexual risk factors. Mdege et al [4] analyzed data from DHS across 28 LMICs, including Zambia, and found that both men and women living with HIV had higher rates of tobacco use including, tobacco smoking, and smokeless tobacco (SLT)(also known as insunko in Zambia) compared to HIV negative counterparts. Similarly, Murphy et al [18] conducted a subsequent analysis across 25 SSA countries, reaffirming that people living with HIV are more likely to use tobacco and smokeless tobacco than their HIV-negative counterparts. More recently study by Kress et al[19]., utilized data from the 2018

Zambia DHS, and reported a higher prevalence of tobacco use among both women and men living with HIV compared to those without HIV. These studies primarily rely on household surveys, which may not capture patterns of tobacco use among PLWH receiving health care services. This underscores the need for facility-based studies to provide more comprehensive insights into tobacco use behaviors, clinical correlates, and targeted intervention opportunities with HIV care settings

To our knowledge, there is a lack of in-depth analyses examining the prevalence of tobacco smoking and smokeless tobacco (SLT) use, along with their associated factors among PLWH receiving regular care in HIV health facilities. This study aims to bridge that gap by estimating the prevalence of tobacco smoking and smokeless tobacco use (SLT) while identifying key predictors influencing these behaviors. Notably, Zambia currently lacks a structured smoking cessation policy for individuals attending routine HIV clinics. The findings from this study can inform the development of targeted smoking cessation interventions, which are critical for reducing the burden of non-communicable diseases (NCDs) and improving overall health outcomes among PLWH

Methodology

Study design and setting

This was a nationally cross-sectional representative survey on non-communicable diseases among PLWH in the 10 provinces of Zambia (figure 1). The survey included HIV+ clients on ART from the health facilities that provide ART services country-wide. Health facilities offering HIV treatment and management services and use the national electronic health record system known as SMARTCARE were included. All HIV+ adults (aged 18 years and above) on ART at the time of the survey and were accessing ART services in the sampled health facility that offers ART services were eligible for enrolment.

Sample size calculation and Enrolment of participants

To estimate the sample size, we used the prevalence formula assuming an infinite population. With no reliable estimate of the prevalence of NCDs among PLWH, we used a prevalence of 50% to estimate the sample size. We estimated a sample size of 385 with a two-sided 95% confidence interval and a 10% margin of error. To ensure adequate representation across the different age-groups (18-29, 30-44, 45-59, and 60+ years), by rural-urban and by sex, we multiplied the calculated sample size by the total number of domains i.e., 8. This yielded a sample of 3,080. To address the issue of clustering and non-response, we multiplied the sample size by a design effect of 1.5 and adjusted for a 20% non-response rate, giving us a total sample size of 5775.

We used a two-stage cluster sampling technique to select a nationally representative sample of health facilities providing ART services. A cluster was defined as a health facility (i.e., clinic) made up of HIV+ patients. Our sampling frame consisted a list of all health facilities providing ART services with a TXR of at least 500 from the electronic health record system (SMARTCARE). In the first sampling stage, facilities were stratified by Province, then secondly by rural-urban creating a total of 20 strata. To allocate the total sample size of 5775 across the 20 strata with a fixed sample take of 35 participants from each health facility, we used probability proportional to size to select 165 health facilities from the sampling frame. Those who were missing outcome variables of interest were excluded from the analysis. Data from the participants was collected between 08/07/2023 to 18/07/2023. To reduce selection bias, four participants were systematically sampled per day, two in the morning and two in the afternoon. Because the main focus of the parent study was NCD’s among PLWH, all pregnant women and breastfeeding mothers were excluded from the study.

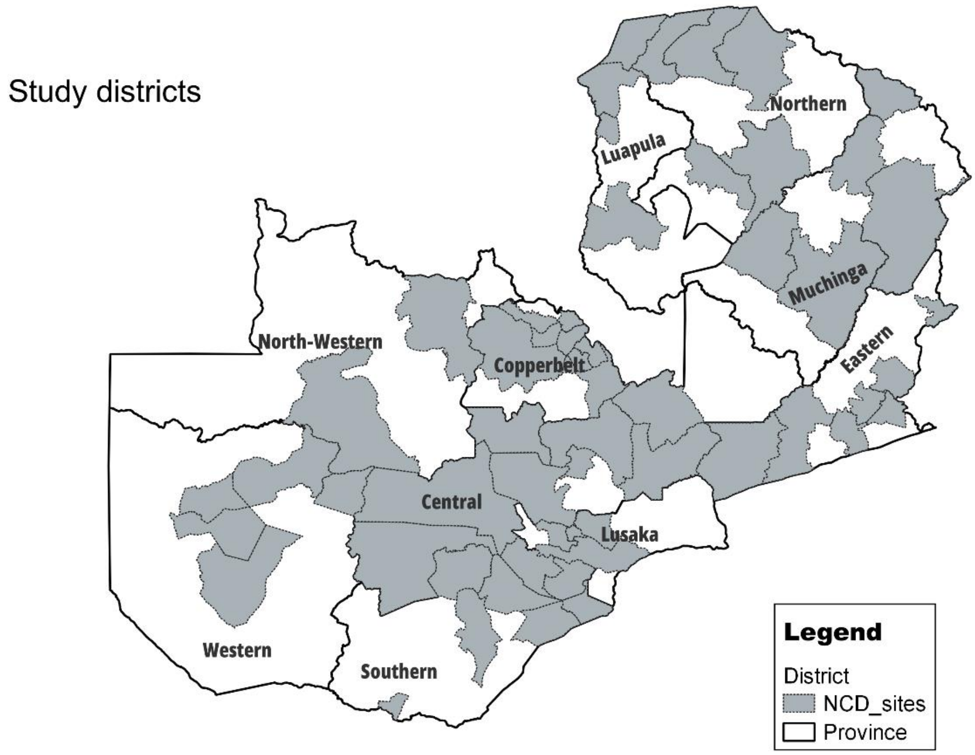

Measures and variables

Outcomes of interest

We have two primary outcomes of interest: 1) Tobacco smoking as asked from the questionnaire “Do you currently smoke any tobacco products, such as cigarettes, Shisha, cigars or pipes?” (Yes/ No); 2) Smokeless tobacco (SLT) use as asked from the questionnaire “Do you currently use any smokeless tobacco products such as *snuff*, *chewing tobacco*?” (Yes/No)

Independent variables

1) **Sociodemographic variables** included sex (male or female) age, education level (No formal schooling, less than primary, Primary school completed, Junior Secondary school completed, Secondary Higher school completed, College/University completed and post graduate degree), Income (< K4000.00 vs ≥ K4000.00); occupation (employed vs non-employed), marital status (single, married or widowed/separated); current smoking status (smoker vs non-smoker)

2) **Behavior variables,** alcohol use as asked "have you consumed any alcohol within the past 30 days?" (Yes or No)

3) **Clinical variables** included diabetes, history of hypertension, history of metabolic diseases (diabetes and dyslipidemia), history of cardiovascular diseases (heart attack, stroke), the question was on each disease "have you ever been told by a doctor or other health worker that you have raised blood pressure, raised blood sugar or diabetes? Raised cholesterol levels? heart attack or stroke? the answer was Yes or No.

Statistical analysis

The characteristics of tobacco smoking and SLT use were analyzed for the overall study population. Continuous variables were summarized using means and Standard Deviations (SD), while categorical variables were presented as frequencies with percentages. To assess associations between exposure variables and tobacco use outcomes, both univariate and multivariable logistic regression models applied. Odds ratios (ORs) and the corresponding 95% confidence intervals (CIs) were calculated to estimate the strength of associations, with adjusted ORs (AORs) accounting for potential confounders. Clinically relevant variables were predetermined and included in the final model regardless of the univariate statistical significance (age, sex, clinical variables) with additional variables included based on a univariate statistical significance ($p<0.05$) with tobacco smoking and SLT status. Statistical

significance level was set at $p<0.05$ (two-tailed test). Analysis was performed using StataCorp. 2017. Stata Statistical Software: Release 15. College Station, TX: StataCorp LLC

Ethical Consideration

Ethics approval was obtained from UNZABREC (REF. 3007-2022). The protocol was subsequently submitted to the Zambia National Health Research Authority (Ref No: NHRA0000011/12/09/2022) for registration and approval. Permission was sought from the Ministry of Health to allow data to be collected from the selected health facilities and for their staff to participate in the study.

Informed consent was secured from all participants before their involvement in the study. We ensured the confidentiality of the data obtained. Each participant provided written informed consent, facilitated by a trained member of the study team. Potential respondents had the opportunity to ask any questions about the research before deciding whether to participate. Those who agreed to participate were interviewed. Participants received written informed consent forms and information sheets detailing the study, its risks and benefits, and emphasizing the protection of confidentiality.

Results

Population and distribution

This study involved 5,204 PLWH who participated in the survey, out of which 3,495 (67.2%) were females and 1709 (32.8%) were males with a median age of 43 (IQR: 34, 51) years. Educational attainment varied, with 2,331 (44.8%) having completed secondary education and 468 (9.0%) having attained higher education levels (i.e., college or university). Most participants were either currently married or cohabiting, accounting for 2,929 (56.3%)

respondents, while the remaining participants had varying marital status. Employment status revealed that 1,041 (20.0%) were formally employed, 1,976 (38.0%) were unemployed, and 2079 (40.0%) were in informal employment. The average yearly income of the participants was categorized into two groups: those earning ≤4,000 ZMW and those earning >4,000 ZMW. 2,389 (45.9%) of the participants fell into the lower income category, while 1,210 (23.3%) were in the higher income category, and 1,605 (30.8%) had missing income data. The study also considered the location of the participants, with 1,627 (31.3%) residing in rural areas and 3,577 (68.7%) in urban areas. Of the 5,204 PLWH, 1756 (33.7%) were either overweight or obese, 872 (18.3%) had a history of Hypertension, 136 (6.5%) raised blood sugar or diabetes, 5 (10.4%) raised cholesterol, 85 (1.6%) heart attack/ stroke and 371(7.1%) were non-adherent to ART (Table 1)

Prevalence and Pattern of tobacco smoking

The overall prevalence of tobacco smoking among PLWH was 503 (9.7%), with a significantly higher proportion of men 375 (74.6%) compared to females 128 (25.4%). The highest smoking prevalence was observed among individuals aged 30-44 (48.9%), those residing in urban areas 335 (66.6%), and individuals with no formal education or only primary education (51.8%). Smoking was also more common among those with Informal (40%), individuals who were currently married or Cohabitating 314 (62.4%), and those with average annual income of ≤4,000 ZMW 256 (51%). Among current smokers 142 (28.2%) were overweight or obese, while 376 (74.8%) reported alcohol consumption in the past 12 months. Regarding clinical conditions, 14.7% of smokers had high blood pressure, 1.2% had a history of a heart attack or stroke, and 1.2% had raised blood sugar levels. Additionally, nearly 16% of tobacco smokers were non-adherent to ART (Table 1). Among tobacco users, manufactured cigarettes were the most commonly used product, with 255(44.7%) smoking daily and 102 (20.3%) smoking

weekly, with a higher prevalence among men. Shisha use was more common on a weekly basis (36%) compared to daily use (29%), with 39.7% of men (n=149) smoking shisha weekly compared to a lower proportion of women (Table 2).

Prevalence and Pattern of smokeless tobacco

The overall prevalence of smokeless tobacco among PLWH was 1.4%, with a higher proportion of women (58.7%) using smokeless tobacco compared to men. The highest prevalence was observed among individuals aged 30-44 (53.3%), those residing in urban areas 57 (76%), and individuals with no formal education or only primary education (57.4%). Smokeless tobacco was also more common among unemployed individuals (39%), those who were married or Cohabitating 40 (53.3%), and those with an annual income of ≤4,000 ZMW 45 (60%). Among current SLT users, 19 (25.3%) were overweight or obese. while 60 (80%) reported consuming alcohol in the last 12 months. Regarding clinical conditions, 24% had high blood pressure (24%), 2.7% had a history of heart attack or stroke, 6.7% had raised blood sugar levels. Additionally, more than 17% of SLT users were non-adherent to ART (Table 1).

Women were more likely than men to use SLT daily, with the most common methods of consumption being snuffing by mouth (43.2%), snuffing by nose (31.8%), and chewing tobacco (31.8%) (Table 2)

Predictors of tobacco smoking and smokeless tobacco

The multivariable analysis identified the factors associated with tobacco smoking among the PLWH. The adjusted odds ratios (AORs) revealed significant associations between gender, education level, alcohol consumption, BMI, and ART adherence. Males had a significantly higher likelihood of smoking compared to females, with an AOR of 4.81 (95% CI: 3.36-6.90). Higher educational attainment was associated with a lower likelihood of smoking with AORs

decreasing as education level increased (primary [AOR 0.81 (0.52-1.26)], secondly [AOR 0.46 (0.30-0.69)], college/university [AOR 0.29 (0.16-0.52)]. Those who consumed alcohol in the past 12 months had increased odds of smoking, with an AOR of 4.97 (2.93-8.44). Overweight or obese participants were less likely to smoke than those with a normal BMI (AORs of 0.55, 95% CI: 0.35-0.85 and AOR 0.36, 95% CI: 0.17-0.79, respectively). Non-adherence to antiretroviral therapy (ART) was associated with higher odds of (AOR of 1.75, 95% CI 1.14-2.67). (Table 3). Additionally, we investigated the factors associated with current SLT. Significant associations were found with income level, alcohol consumption, and ART adherence. Participants with an annual income of ≤K4,000 were more likely to use smokeless tobacco compared to those earning >K4,000 (AOR of 0.31, 95% CI: 0.14-0.73). Those who consumed alcohol in the past 12 months had significantly higher odds of using smokeless tobacco, (AOR 14.74, 95% CI: 1.99-109.02). Non-adherence to ART was associated with a higher likelihood of SLT use (AOR of 3.32, 95% CI: 1.54-7.17) (Table 4).

Discussion

To our knowledge this is the first study that has estimated the prevalence of tobacco smoking; and smokeless tobacco (SLT) and determined the socio-demographic, clinical and behavioral characteristics among the PLWH receiving routine clinical care in Zambia. The study has shown that the prevalence of tobacco smoking and smokeless tobacco smoking is 9.7% and 1.4% respectively. Males smoked tobacco more than they used smokeless tobacco, contrary to women who used smokeless tobacco than tobacco smoking. Being male, having secondary level of education, alcohol consumption, being overweight/obese and non-adherence to ART were associated with the likelihood to smoking tobacco, while income of the patient, alcohol consumption and non-adherence to ART were associated with a higher likelihood to SLT.

The prevalence of the PLWH who smoke tobacco and receiving routine clinical care is 9.7% surprisingly, its lower than what is recorded in previous studies of the same settings [5,10,20]. Generally, literature has shown that PLWH use tobacco products two to three times more than the general population [19,21,22], this was our expectation, however, the prevalence in PLWH in our study did not show a difference as compared to the general population, this is unexpected. This could mainly be under reporting due to social desirability bias. When the population was categorized by gender, it was not surprising to note that our study showed a higher prevalence of tobacco smoking in men (21.9%) compared to women (3.7%). This conforms with previous HIV/tobacco studies. A recent HIV/tobacco study by Mdege et al. using the Demographic Health Survey (DHS)[4] showed that 24.4% and 1.3% of men and women smoked tobacco, respectively. Similarly, Kress et al[19] using DHS showed that 19.5% and 2.7% of men and women smoked tobacco, respectively. From these two recent studies, our study has revealed a concerning rise in the prevalence of tobacco smoking among women, marking a significant public health issue. Regarding smokeless tobacco, it's not surprising to see that the proportion of WLWH who use smokeless tobacco is higher than that of men. This conforms with previous studies.[23,24].

After adjusting for socio - demographic and clinical factors, our analyses on both tobacco smoking and SLT model showed that men were significantly more likely to smoke tobacco than women; and women were more likely to use SLT than men although this could not reach statistical significancy. The proportion of those using SLT our study was low (1.4%), limiting our ability to make broad inferences about this segment of our population. Over the past two decades, research has highlighted the influence of gender-related factors on smoking behavior[17,25]. Pampel et al[26] examined population-based data from 16 DHS of men aged 15-54

years and women aged 15-49 years in 14 sub-Saharan countries and showed that although tobacco use varies widely across sub-Saharan African countries, one consistent pattern is that smoking rates are higher among men than women. Social norms and taboos play a significant role. Women face stronger cultural restrictions against smoking, which contributes to their lower prevalence. In contrast to SLT, women tend to use SLT more than men, this could be due to the myths and perceptions hence not surprising to see that woman living with HIV (WLWH) used SLT more than men in our study. Our findings are not different from other studies in the region[23]. What's of public health concern is that more than half of the WLWH in our study used SLT. The plausible explanation could be that its culturally acceptable mode of using tobacco in women. It's also could be driven mainly by these strong myths and perceptions that smokeless tobacco particularly nsunko enhances sexual enjoyment as it dries and makes the vaginal tight; and that it's an immunity booster and aid in viral suppression in PLWH.

Educational level, and income levels have been used as an individual's economic status, each one of them using a different causal pathway to explain the health outcomes[27]. Our study revealed a notable inverse relationship between education level and tobacco smoking. Smokeless tobacco though showed the same association, it was not statistically significant. As an individual's education level increases, the likelihood of tobacco smoking decreases. This finding aligns with prior research conducted in both developed and developing countries[28-30]. Education plays a crucial role in shaping health behavior decisions in that individuals with higher education tend to make more informed choices leading to better health outcomes. As regards to income, only the regression model for smokeless tobacco use showed a statistical significancy. Those with lower income ≤4,000 ZMW were more likely to use SLT than those with >4,000 ZMW. A plethora of literature has shown that prevalence smoking and tobacco

use is highest in the lower income as compared the high income[30,31]. Nsunko sold in sachets is cheap and affordable, women can easily access the product even within the neighborhood.

Numerous studies have demonstrated a link between alcohol consumption and tobacco smoking [32,33], it's not surprising that almost 75% of the tobacco smokers and 80% of SLT users reported alcohol consumption. Our study showed correlation between alcohol consumption and tobacco smoking; between alcohol consumption and SLT, with those who consume alcohol being almost 5 times more likely to smoke tobacco while being almost 14 times more likely to use SLT. Several mechanisms may contribute to the strong association between alcohol consumption and tobacco use, Swan et al, propose that genetic factors contribute significantly to the covariance of both behaviors[34], while Niaura et al, advances the stress-coping theory that suggests that alcohol and tobacco serve as coping mechanisms to regulate emotions[35].

In conformity with other studies [36-38], our study also showed that there a significant association between underweight /overweight/obesity and tobacco smoking. Tobacco smokers weigh less than the non-smokers because the nicotine affects weight through biological and behavior mechanisms[36,37,39]. The physiological effect of nicotine is that it results in appetite suppression, increase resting metabolism rates and reduced calorie storage[40-42]. Smoking is also related to decrease to meal size[40], and some of the smokers use smoking as a marker for meal termination in that instead of eating they would substitute smoking[42]. This association was not noted in PLWH who use smokeless tobacco. Our study has showed that the PWLH who smoke tobacco and use smokeless tobacco are less likely to adhere to ART. Previous studies have indicated that ART adherence in PLWH is a significant problem[43] but more so in PLWH who smoke[44,45]. Aggarwal et al. have reported that smokers are less adherent to other medications as well[46]. A potential explanation may be that smoke tobacco and use smokeless tobacco are

less likely to engage in self-care behaviors such as ART adherence compared with non-smokers.

Our study is not without some limitations. Due to the observational design, we were unable to assess changes in these factors over time. This design allowed us to identify factors associated with tobacco smoking and smokeless tobacco use but did not permit us to establish causality. Although we adjusted for known confounders in the multivariable model, the potential for residual confounders inherent in observational studies remains, which might affect the interpretation of our findings. The data is based on self-reported responses, which may introduce information bias due to the misclassification of tobacco smoking and smokeless tobacco use. Additionally, the generalizability of our findings is limited as this was a single cross-sectional study conducted in Zambia. To our knowledge, this is the first study in Zambia to determine national tobacco smoking and smokeless tobacco use among PLWH. It had a large sample size and was weighted based on the HIV burden at the district level. Surveyed PLWH were randomly selected from well-established ART clinics across the country, making the results generalizable to the general HIV population in Zambia.

In conclusion, our study found that, being a male, with lower education, consuming alcohol and non-adherent to ART is associated to tobacco smoking; while being a female, with an average yearly income of ≤4,000 ZMW and non-adherent to ART is associated to SLT. Our study underscores the need for specific interventions identified to reduce tobacco use. Firstly, develop gender-specific strategies that address the unique factors influencing tobacco smoking and SLT use among males and females respectively. For instance, for females with an annual income of ≤4,000 ZMW, focus on economic empowerment and support to improve their financial situation and reduce reliance on smokeless tobacco (SLT). Additionally, provide support for individuals who are non-adherent to ART. This support could involve regular

follow-ups and personalized adherence plans. Moreover, implement programs that address alcohol consumption, as it is associated with tobacco smoking, including counselling and support groups.

Acknowledgments

We extend our deepest appreciation to all individuals and institutions whose support and contributions were critical to the successful implementation of this project. This endeavor would not have been possible without their invaluable assistance.

We thank the Ministry of Health and the Global Fund through the Prevention of HIV, TB, and Malaria for their financial and technical support. We are also profoundly grateful to the Ministry of Health Leadership, particularly the Non-Communicable Disease Unit, for their oversight and guidance throughout the study. Their expertise and support were essential in maintaining the highest standards of ethics and safety.

Additionally, we offer heartfelt thanks to the research assistants for their valuable contributions to data collection and analysis, which were fundamental to the project's success. We also acknowledge all the investigators for their crucial roles, providing expertise, guidance, and support pivotal to this study. We also extend our gratitude the project management team for their tireless efforts in overseeing daily activities, ensuring efficient planning and execution within the stipulated timeframe and budget. Finally, we express special thanks to all participants who agreed to take part in the study without them this project was not going to be successful.

References

1. Gandhi RT, Bedimo R, Hoy JF, et al. Antiretroviral Drugs for Treatment and Prevention of HIV Infection in Adults: 2022 Recommendations of the International Antiviral Society-USA Panel. *JAMA.* Jan 3 2023;329(1):63-84.
2. UNAIDS. In Danger: UNAIDS Global AIDS Update 2022 *UNAIDS (https://www.unaids.org/en/resources/documents/2022/in-danger-global-aids-update) accessed 10_08_24.* 2022.
3. Kharsany AB, Karim QA. HIV Infection and AIDS in Sub-Saharan Africa: Current Status, Challenges and Opportunities. *Open AIDS J.* 2016;10:34-48.
4. Mdege ND, Shah S, Ayo-Yusuf OA, Hakim J, Siddiqi K. Tobacco use among people living with HIV: analysis of data from Demographic and Health Surveys from 28 low-income and middle-income countries. *Lancet Glob Health.* Jun 2017;5(6):e578-e592.
5. Zyambo CM, Willig JH, Cropsey KL, et al. Factors Associated With Smoking Status among HIV-Positive Patients in Routine Clinical Care. *J AIDS Clin Res.* Jul 2015;6(7).
6. Tesoriero JM, Gieryic SM, Carrascal A, Lavigne HE. Smoking among HIV positive New Yorkers: prevalence, frequency, and opportunities for cessation. *AIDS and Behavior.* 2010;14:824-835.
7. Shamo F MK, Hitchingham J, Bennett S, Watson S. The Effect of a Tobacco Use Reduction Program on the Prevalence of Smoking and Tobacco Use and Quitting Behavior Among People Living With HIV/AIDS in Michigan. *Precenting Chronic Disease* 2024;21(230115).
8. Adhikari B, Kahende J, Malarcher A, Pechacek T, Tong V. Smoking-attributable mortality, years of potential life lost, and productivity losses-United States, 2000-2004. 2008.

9. Nguyen NT, Tran BX, Hwang LY, et al. Effects of cigarette smoking and nicotine dependence on adherence to antiretroviral therapy among HIV-positive patients in Vietnam. *AIDS Care.* 2016;28(3):359-364.
10. Tumwegamire A, Fatch R, Emenyonu NI, et al. Association between smoking and lack of HIV virological suppression in a cross-sectional study of persons with HIV on antiretroviral therapy in Uganda. *PLoS One.* 2024;19(3):e0300508.
11. Hile SJ, Feldman MB, Alexy ER, Irvine MK. Recent Tobacco Smoking is Associated with Poor HIV Medical Outcomes Among HIV-Infected Individuals in New York. *AIDS and Behavior.* 2016/08/01 2016;20(8):1722-1729.
12. Crothers K, Goulet JL, Rodriguez-Barradas MC, et al. Impact of cigarette smoking on mortality in HIV-positive and HIV-negative veterans. *AIDS Education and Prevention.* 2009;21(3_supplement):40-53.
13. Ministry of Health Z. *Zambia Population-based HIV Impact Assessment (ZAMPHIA) 2021: Final Report. Lusaka, Ministry of Health, DECEMBER 2023*2023.
14. Muyinza FO, P Michelo, C. Tobacco smoking prevalent in Zambian males: Observations from the Zambia Demographic Health Survey 2013-2014. *Medical Journal of Zambia.* 2021;47(3).
15. Zambia Statistics Agency MoH, and ICF. *Zambia Demographic and Health Survey 2018. Lusaka, Zambia, and Rockville, Maryland, USA: Zambia Statistics Agency, Ministry of Health, and ICF.*2019.
16. Zyambo C, Olowski P, Mulenga D, Liamba F, Syapiila P, Siziya S. School tobacco-related curriculum and behavioral factors associated with cigarette smoking among school-going adolescents in Zambia: Results from the 2011 GYTS study. *Tob Induc Dis.* 2022;20:42.

17. Zyambo C, Olowski P, Mulenga D, et al. Prevalence and Factors Associated with Tobacco Smoking in a National Representative Sample of Zambian Adults: Data from the 2017 STEPS - NCDs Survey. *Asian Pac J Cancer Prev.* Jan 1 2023;24(1):111-119.

18. Murphy JD, Liu B, Parascandola M. Smoking and HIV in Sub-Saharan Africa: A 25-Country Analysis of the Demographic Health Surveys. *Nicotine Tob Res.* Jul 17 2019;21(8):1093-1102.

19. Kress AC, Stadnik C, Phiri MM, Goma FM, Twentyman E. Tobacco Use among HIV-Positive and HIV-Negative Women and Men in Zambia-Demographic and Health Survey, 2018. *Int J Environ Res Public Health.* Mar 24 2022;19(7).

20. Shamo F, Macomber KE, Hitchingham J, Bennett S, Watson S. The Effect of a Tobacco Use Reduction Program on the Prevalence of Smoking and Tobacco Use and Quitting Behavior Among People Living With HIV/AIDS in Michigan. *Prev Chronic Dis.* Jan 11 2024;21:E03.

21. Drach L, Holbert T, Maher J, Fox V, Schubert S, Saddler LC. Integrating smoking cessation into HIV care. *AIDS Patient Care STDS.* Mar 2010;24(3):139-140.

22. Mamary EM, Bahrs D, Martinez S. Cigarette smoking and the desire to quit among individuals living with HIV. *AIDS Patient Care STDS.* Jan 2002;16(1):39-42.

23. Loh SM, Moloantoa T, Yende N, et al. A cross-sectional study of attitudes and behaviors of snuff use and cessation among people with and without HIV in South Africa. *Tobacco Use Insights.* 2023/01/01 2023;16:1179173X231193890.

24. Elf JL, Variava E, Chon S, et al. Prevalence and Correlates of Snuff Use, and its Association With Tuberculosis, Among Women Living With HIV in South Africa. *Nicotine Tob Res.* Jul 17 2019;21(8):1087-1092.

25. Nyirenda HT, Mulenga D, Silitongo M, Nyirenda HB, Nyirenda T. Spatial distribution and correlates of smoking in Zambia. *BMJ Open.* Aug 10 2019;9(8):e030044.

26. Pampel F. Tobacco use in sub-Sahara Africa: estimates from the demographic health surveys. *Soc Sci Med.* Apr 2008;66(8):1772-1783.

27. Blakely T, Wilson N. The contribution of smoking to inequalities in mortality by education varies over time and by sex: two national cohort studies, 1981–84 and 1996–99. *International Journal of Epidemiology*. 2005;34(5):1054-1062.

28. Cavelaars AE, Kunst AE, Geurts JJ, et al. Educational differences in smoking: international comparison. *BMJ.* Apr 22 2000;320(7242):1102-1107.

29. Pampel FC. Global Patterns and Determinants of Sex Differences in Smoking. *Int J Comp Sociol.* Dec 2006;47(6):466-487.

30. Leinsalu M, Kaposvári C, Kunst AE. Is income or employment a stronger predictor of smoking than education in economically less developed countries? A cross-sectional study in Hungary. *BMC Public Health.* 2011/02/13 2011;11(1):97.

31. Casetta B, Videla AJ, Bardach A, et al. Association Between Cigarette Smoking Prevalence and Income Level: A Systematic Review and Meta-Analysis. *Nicotine Tob Res.* Nov 7 2017;19(12):1401-1407.

32. Cosmas Zyaambo OB PS, Adamson S Muula, Emmanuel Rudatsikira, Seter Siziya. Alcohol Consumption and its Correlates Among Residents of Mining Town, Kitwe, Zambia: 2011 Population Based Survey. *American Medical Journal.* 2013;4(1):6-11.

33. Friedman GD, Tekawa I, Klatsky AL, Sidney S, Armstrong MA. Alcohol drinking and cigarette smoking: an exploration of the association in middle-aged men and women. *Drug Alcohol Depend.* May 1991;27(3):283-290.

34. Swan GE, Carmelli D, Rosenman RH, Fabsitz RR, Christian JC. Smoking and alcohol consumption in adult male twins: genetic heritability and shared environmental influences. *J Subst Abuse.* 1990;2(1):39-50.

35. Niaura R. SSFJB AJP. Alcohol and Tobacco: From Basic Science to Clinical Practice. . *Washington, DCUnited States Department of Health and Human Services(pg. 159-168)NIH Publication 95-397 Psychosocial and biological mechanism.* 1995:159-168.

36. Albanes D, Jones DY, Micozzi MS, Mattson ME. Associations between smoking and body weight in the US population: analysis of NHANES II. *Am J Public Health.* Apr 1987;77(4):439-444.

37. Canoy D, Wareham N, Luben R, et al. Cigarette smoking and fat distribution in 21,828 British men and women: a population-based study. *Obes Res.* Aug 2005;13(8):1466-1475.

38. Yore MM, Fulton JE, Nelson DE, Kohl HW, III. Cigarette Smoking Status and the Association between Media Use and Overweight and Obesity. *American Journal of Epidemiology.* 2007;166(7):795-802.

39. Rásky E, Stronegger WJ, Freidl W. The relationship between body weight and patterns of smoking in women and men. *Int J Epidemiol.* Dec 1996;25(6):1208-1212.

40. Jo YH, Talmage DA, Role LW. Nicotinic receptor-mediated effects on appetite and food intake. *J Neurobiol.* Dec 2002;53(4):618-632.

41. Perkins KA. Metabolic effects of cigarette smoking. *J Appl Physiol (1985).* Feb 1992;72(2):401-409.

42. Wack JT, Rodin J. Smoking and its effects on body weight and the systems of caloric regulation. *Am J Clin Nutr.* Feb 1982;35(2):366-380.

43. Levine AJ, Hinkin CH, Marion S, et al. Adherence to antiretroviral medications in HIV: differences in data collected via self-report and electronic monitoring. *Health Psychol.* May 2006;25(3):329-335.

44. King RM, Vidrine DJ, Danysh HE, et al. Factors associated with nonadherence to antiretroviral therapy in HIV-positive smokers. *AIDS Patient Care STDS.* Aug 2012;26(8):479-485.

45. O'Cleirigh C, Valentine SE, Pinkston M, et al. The unique challenges facing HIV-positive patients who smoke cigarettes: HIV viremia, ART adherence, engagement in HIV care, and concurrent substance use. *AIDS Behav.* Jan 2015;19(1):178-185.

46. Aggarwal B, Mosca L. Lifestyle and psychosocial risk factors predict non-adherence to medication. *Ann Behav Med.* Oct 2010;40(2):228-233.

Table 1: Proportions of Sociodemographic, Behavioural, and Clinical Variables (Tobacco Smoking and Smokeless Tobacco)

	Overall	Smoke tobacco		Smokeless tobacco		
	N (% of total)	No n (%)	Yes n (%)	No n (%)	Yes n (%)	
Characteristics	5204 (100)	4701 (90.3)	503 (9.7)	5129 (98.6)	75 (1.4)	
**Gender**						
Female	3495 (67.2)	3367 (71.6)	128 (25.4)	3451 (67.3)	44 (58.7)	
Male	1709 (32.8)	1334 (28.4)	375 (74.6)	1678 (32.7)	31 (41.3)	
**Age (years)**						
median (IQR)						
18-29	724 (13.9)	676 (14.4)	48 (9.5)	718 (14.0)	6 (8.0)	
30-44	2202 (42.3)	1956 (41.6)	246 (48.9)	2162 (42.2)	40 (53.3)	
45-59	1836 (35.3)	1661 (35.3)	175 (34.8)	1811 (35.3)	25 (33.3)	
60+	422 (8.1)	391 (8.3)	31 (6.2)	419 (8.2)	3 (4.0)	
Missing	20 (0.4)	17 (0.4)	3 (0.6)	19 (0.4)	1 (1.3)	
**Education**						
No formal schooling	1131 (21.7)	1002 (21.3)	129 (25.6)	1108 (21.6)	23 (30.7)	
Primary	1271 (24.4)	1139 (24.2)	132 (26.2)	1251 (24.4)	20 (26.7)	
Secondary	2331 (44.8)	2118 (45.1)	213 (42.3)	2301 (44.9)	30 (40.0)	
College/University	468 (9.0)	439 (9.3)	29 (5.8)	466 (9.1)	2 (2.7)	
Missing	3 (0.1)	3 (0.1)	0 (0.0)	3 (0.1)	0 (0.0)	
**Marital status**						
Never married	705 (13.5)	639 (13.6)	66 (13.1)	696 (13.6)	9 (12.0)	
Currently married/Cohabitating	2929 (56.3)	2615 (55.6)	314 (62.4)	2889 (56.3)	40 (53.3)	
Divorced/Separated/Widowed	1551 (29.8)	1430 (30.4)	121 (24.1)	1525 (29.7)	26 (34.7)	
Missing	19 (0.4)	17 (0.4)	2 (0.4)	19 (0.4)	0 (0.0)	
**Employment status**						
Formal employment	1041 (20.0)	931 (19.8)	110 (21.9)	1023 (19.9)	18 (24.0)	
Informal employment	2079 (40.0)	1861 (39.6)	218 (43.3)	2053 (40.0)	26 (34.7)	
Unemployed	1976 (38.0)	1808 (38.5)	168 (33.4)	1947 (38.0)	29 (38.7)	
Missing	108 (2.1)	101 (2.1)	7 (1.4)	106 (2.1)	2 (2.7)	
**Average yearly income**						
≤4,000 ZMW	2389 (45.9)	2133 (45.4)	256 (50.9)	2344 (45.7)	45 (60.0)	
>4,000 ZMW	1210 (23.3)	1101 (23.4)	109 (21.7)	1202 (23.4)	8 (10.7)	
Missing	1605 (30.8)	1467 (31.2)	138 (27.4)	1583 (30.9)	22 (29.3)	
**Location**						

Rural	1627 (31.3)	1459 (31.0)	168 (33.4)	1609 (31.4)	18 (24.0)	
Urban	3577 (68.7)	3242 (69.0)	335 (66.6)	3520 (68.6)	57 (76.0)	
**Behavior variables**						
**Consumed alcohol within past 12m***						
No	494 (24.4)	471 (10.0)	23 (4.6)	492 (9.6)	2 (2.7)	
Yes	1527 (75.6)	1151 (24.5)	376 (74.8)	1467 (28.6)	60 (80.0)	
**Clinical variables**						
**BMI**						
Normal weight (18.5-24.9)	2915 (56.0)	2578 (54.8)	337 (67.0)	2866 (55.9)	49 (65.3)	
Underweight (≤18.4)	453 (8.7)	374 (8.0)	79 (15.7)	444 (8.7)	9 (12.0)	
Overweight (25-29.9)	1166 (22.4)	1103 (23.5)	63 (12.5)	1156 (22.5)	10 (13.3)	
Obese (≥30)	590 (11.3)	570 (12.1)	20 (4.0)	585 (11.4)	5 (6.7)	
Missing	80 (1.5)	76 (1.6)	4 (0.8)	78 (1.5)	2 (2.7)	
**Told by a doctor or other health worker that you have raised BP or HTN***						
No	3883 (81.7)	3507 (74.6)	376 (74.8)	3833 (74.7)	50 (66.7)	
Yes	872 (18.3)	798 (17.0)	74 (14.7)	854 (16.7)	18 (24.0)	
**Told by a doctor or other health worker that you have raised blood sugar or diabetes***						
No	1952 (93.5)	1775 (37.8)	177 (35.2)	1922 (37.5)	30 (40.0)	
Yes	136 (6.5)	130 (2.8)	6 (1.2)	131 (2.6)	5 (6.7)	
**Told by a doctor or other health worker that you have raised cholesterol***						
No	43 (89.6)	42 (0.9)	1 (0.2)	42 (0.8)	1 (1.3)	
Yes	5 (10.4)	5 (0.1)	0 (0.0)	5 (0.1)	0 (0.0)	
**Heart attack or stroke**						
No	5119 (98.4)	4622 (98.3)	497 (98.8)	5046 (98.4)	73 (97.3)	
Yes	85 (1.6)	79 (1.7)	6 (1.2)	83 (1.6)	2 (2.7)	
**ART adherence status**						
Adherent	4833 (92.9)	4410 (93.8)	423 (84.1)	4771 (93.0)	62 (82.7)	
Non-adherent	371 (7.1)	291 (6.2)	80 (15.9)	358 (7.0)	13 (17.3)	

Table 2: Patterns of Tobacco Use Among Participants, Stratified by Type (Smoke and Smokeless)

Types of Tobacco	Overall<br>n<br>(% of total)	Sex<br>Female<br>n (%)	 Male<br>n (%)
**Smoke Tobacco**			
**Manufactured cigarettes**			
Daily	225 (44.7)	28 (21.9)	197 (52.5)
Weekly	102 (20.3)	27 (21.1)	75 (20.0)
No	176 (35.0)	73 (57.0)	103 (27.5)
**Hand rolled cigarettes**			
Daily	174 (34.6)	23 (18.0)	151 (40.3)
Weekly	153 (30.4)	32 (25.0)	121 (32.3)
No	176 (35.0)	73 (57.0)	103 (27.5)
**Pipes full of tobacco**			
Daily	162 (32.2)	23 (18.0)	139 (37.1)
Weekly	165 (32.8)	32 (25.0)	133 (35.5)
No	176 (35.0)	73 (57.0)	103 (27.5)
**Cigars, cheroots or cigarillos**			
Daily	159 (31.6)	23 (18.0)	136 (36.3)
Weekly	168 (33.4)	32 (25.0)	136 (36.3)
No	176 (35.0)	73 (57.0)	103 (27.5)
**Shisha**			
Daily	146 (29.0)	23 (18.0)	123 (32.8)
Weekly	181 (36.0)	32 (25.0)	149 (39.7)
No	176 (35.0)	73 (57.0)	103 (27.5)
**Total**	**503 (100)**	**128 (25.5)**	**375 (74.5)**

**Smokeless tobacco**			
**Snuff, by mouth**			
Daily	26 (34.7)	19 (43.2)	7 (22.6)
Weekly	5 (6.7)	3 (6.8)	2 (6.5)
No	44 (58.7)	22 (50.0)	22 (71.0)
**Snuff by nose**			
Daily	21 (28.0)	14 (31.8)	7 (22.6)
Weekly	10 (13.3)	8 (18.2)	2 (6.5)
No	44 (58.7)	22 (50.0)	22 (71.0)
**Chewing tobacco**			
Daily	19 (25.3)	14 (31.8)	5 (16.1)
Weekly	12 (16.0)	8 (18.2)	4 (12.9)
No	44 (58.7)	22 (50.0)	22 (71.0)
**Betel, quid with tobacco**			
Daily	15 (20.0)	10 (22.7)	5 (16.1)
Weekly	16 (21.3)	12 (27.3)	4 (12.9)
No	44 (58.7)	22 (50.0)	22 (71.0)
**Total**	**75 (100)**	**44 (58.7)**	**31 (41.3)**

Table 3: Factors Associated with tobacco smoking Among PLHIV

Characteristics	Crude OR (95% CI)	chi2 Statistic	p-value	Adjusted OR (95% CI)	p-value
**Gender**					
Female	Ref	410.00	<0.001	Ref	**<0.001**
Male	7.39 (5.99-9.13)			4.81 (3.36-6.9)	
**Age (years)**					
18-29	Ref	16.82	0.001	Ref	0.080
30-44	1.77 (1.28-2.44)			1.48 (0.85-2.57)	
45-59	1.48 (1.06-2.07)			0.99 (0.56-1.78)	
60+	1.12 (0.70-1.78)			0.91 (0.42-1.98)	
**Education**					
No formal schooling	Ref	12.52	0.006	Ref	**<0.001**
Primary	0.90 (0.70-1.16)			0.81 (0.52-1.26)	
Secondary	0.78 (0.62-0.98)			0.46 (0.30-0.69)	
College/University	0.51 (0.34-0.78)			0.29 (0.16-0.52)	
**Marital status**					
Never married	Ref	10.26	0.006		
Currently married/Cohabitating	1.16 (0.88-1.54)				
Divorced/Separated/Widowed	0.82 (0.60-1.12)				
**Employment status**					
Formal employment	Ref	5.66	0.059		
Informal employment	0.99 (0.78-1.26)				
Unemployed	0.79 (0.61-1.01)				
**Average yearly income**					
≤4,000 ZMW	Ref	2.62	0.106	Ref	0.110
>4,000 ZMW	0.82 (0.65-1.04)			0.76 (0.55-1.06)	
**Location**					
Rural	Ref	1.17	0.280		
Urban	0.90 (0.74-1.09)				
**Behavior variables**					
**Consumed alcohol within past 12m**					
No	Ref	117.51	<0.001	Ref	**<0.001**
Yes	6.69 (4.33-10.33)			4.97 (2.93-8.44)	
**Clinical variables**					
**BMI**					
Normal weight (18.5-24.9)	Ref	100.35	<0.001	Ref	**0.001**
Underweight (≤18.4)	1.62 (1.24-2.11)			1.45 (0.93-2.26)	
Overweight (25-29.9)	0.44 (0.33-0.58)			0.55 (0.35-0.85)	
Obese (≥30)	0.27 (0.17-0.43)			0.36 (0.17-0.79)	
**Told by a doctor or other health worker that you have raised BP or HTN****					
No	Ref	1.22	0.269	Ref	0.870
Yes	0.86 (0.67-1.12)			0.97 (0.64-1.45)	

**Told by a doctor or other health worker that you have raised blood sugar or diabetes**					
No	Ref	4.10	0.043		
Yes	0.46 (0.20-1.06)				
**Heart attack or stroke**					
No	Ref	0.73	0.391	Ref	0.22
Yes	0.71 (0.31-1.63)			0.49 (0.15-1.54)	
**ART adherence status**					
Adherent	Ref	50.95	<0.001	Ref	
Non-adherent	2.87 (2.19-3.74)			1.75 (1.14-2.67)	**0.010**

Table 4: Factors Associated with Smokeless Tobacco Use Among PLHIV

Characteristics	Crude OR (95% CI)	chi2 Statistic	p-value	Adjusted OR (95% CI)	p-value
**Gender**					
Female	Ref	2.40	0.121	Ref	0.287
Male	1.45 (0.91-2.30)			0.70 (0.36-1.36)	
**Age (years)**					
18-29	Ref	6.30	0.098	Ref	0.698
30-44	2.21 (0.93-5.24)			1.71 (0.49-5.91)	
45-59	1.65 (0.67-4.04)			1.47 (0.40-5.42)	
60+	0.86 (0.21-3.44)			0.67 (0.07-6.86)	
**Education**					
No formal schooling	Ref	7.71	0.052		
Primary	0.77 (0.42-1.41)				
Secondary	0.63 (0.36-1.09)				
College/University	0.21 (0.05-0.88)				
**Marital status**					
Never married	Ref	0.83	0.660		
Currently married/Cohabitating	1.07 (0.52-2.22)				
Divorced/Separated/Widowed	1.32 (0.61-2.83)				
**Employment status**					
Formal employment	Ref	1.13	0.567		
Informal employment	0.72 (0.39-1.32)				
Unemployed	0.85 (0.47-1.53)				
**Average yearly income**					
≤4,000 ZMW	Ref	9.46	0.002	Ref	**0.007**
>4,000 ZMW	0.35 (0.16-0.74)			0.31 (0.14-0.73)	
**Location**					
Rural	Ref	1.96	0.161		
Urban	1.45 (0.85-2.47)				
**Behavior variables**					
**Consumed alcohol within past 12m***					
No	Ref	22.07	<0.001	Ref	**0.008**
Yes	10.06 (2.45-41.31)			14.74 (1.99-109.02)	
**Clinical variables**					
**BMI**					
Normal weight (18.5-24.9)	Ref	6.94	0.074		
Underweight (≤18.4)	1.19 (0.58-2.43)				
Overweight (25-29.9)	0.51 (0.26-1.00)				
Obese (≥30)	0.5 (0.20-1.26)				
**Told by a doctor or other health worker that you have raised BP or HTN****					
No	Ref	2.77	0.096	Ref	0.122
Yes	1.62 (0.94-2.78)			1.81 (0.85-3.85)	
**Told by a doctor or other health worker that you have raised blood sugar or diabetes*****					
No	Ref	2.71	0.100		
Yes	2.45 (0.93-6.41)				

**Heart attack or stroke**					
No	Ref	0.43	0.514	Ref	0.851
Yes	1.67 (0.40-6.9)			1.22 (0.16-9.46)	
**ART adherence status**					
Adherent	Ref	8.84	0.003	Ref	**0.002**
Non-adherent	2.79 (1.52-5.13)			3.32 (1.54-7.17)	

Click here to access/download
Supporting Information
Ethical approval - NCD_HIV.pdf

Click here to access/download
Supporting Information
NHRA - Approved.pdf
