# Supplementary material for: Tobacco smoking and smokeless tobacco use among people living with HIV in Zambia: Findings from a 2023 National NCD/HIV Survey
Source: PLoS One. 2025 Jul 14;20(7):e0327130. doi: 10.1371/journal.pone.0327130 (PMC12258560; doi:10.1371/journal.pone.0327130)
Supplement: Attachment — Submitted filename: Review PLOS_2.docx [file pone.0327130.s002.docx]

I would like to congratulate the authors on this fine piece of work, exploring an important public health issue in SSA. I would recommend a major revision of the manuscript before acceptance.

In line 120, the authors mentioned that “Because the main focus of the parent study was NCD’s among PLWH, all pregnant women and breastfeeding mothers were excluded from the study”. Please kindly explain the rationale of including age group of 18-29 years old.

Sampling yielded a result of 67.2% females and 32.8% males PLWH (line 182). Is this generally representative of the statistics of PLWH living in the community among SSA? If this is not so, please explain why you think the study results deviate from statistics in the community. Please do input these thoughts under discussion.

In line 267, the authors mentioned that the unexpected finding was due to under reporting due to social desirability bias which could be the case. What is another possible explanation for this difference?

In lines 355-363, the authors should consider rephrasing the sentences, paying attention to sentence structuring and how future direction should be undertaken with respect to the study findings. Do take note that the associations are not causal as the authors have rightly pointed out. Henceforth, one should be careful in mentioning the intervention and associating them with subsequent reduction in tobacco use. For example, does improving financial means truly reduce reliance on smokeless tobacco use or is education an intermediary between financial situation and tobacco use?
